# Gender-based violence – An increasing epidemic in South Africa

**DOI:** 10.4102/safp.v65i1.5729

**Published:** 2023-03-31

**Authors:** Indiran Govender

**Affiliations:** 1 Department of Family Medicine and Primary Health Care, Faculty of Health Sciences, Sefako Makgatho Health Sciences University, Pretoria, South Africa

February is commonly referred to as the month of love but for many women, February will continue to be a dreadful and distressing period with one woman being raped every 3 h in South Africa (SA). South Africa is considered to be the rape capital of the world with 10 818 rape cases reported in the first quarter of 2022.^1^ The rate at which women are killed by intimate partners in this country is five times higher than the global average.^2^ Gender-based violence (GBV), a widespread and common occurrence in SA, is deeply ingrained in homes, workplaces, cultures and traditions. This pandemic, because of unequal power between genders, has far-reaching effects that go on beyond the violence itself. Gender-based violence manifests in various forms that include physical, emotional, psychological, financial or structural harm usually perpetrated by intimate partners, work colleagues, strangers and even institutions.^3^ However, documenting, reporting, intervening and preventing GBV is currently a major health challenge.^2^ Gender-based violence is recognised by the World Health Organization as a major public health problem. Not only is it a direct cause of injury, morbidity and death, but women’s health is affected indirectly through unwanted pregnancies and accompanying health risks, as well as mental illness, sexually transmitted diseases, human immunodeficiency virus (HIV) and acquired immunodeficiency syndrome (AIDS).^2,4^

In many areas of South Africa, there is limited access to formal psychosocial or medical support for survivors of GBV contributing to psychological trauma and behavioural consequences with an inability to reintegrate into society.^2,4^

The HIV and AIDS epidemic in sub-Saharan region of Africa is directly related to domestic violence and sexual violence, making women and young girls in particular vulnerable to HIV. The power structures that dominate society and perpetuate economic, social and cultural inequalities that place women in subordinate positions with regard to meeting their basic needs, protecting their bodies, participating and making decisions in society contribute significantly to this global pandemic.^2,4,5^

Why is GBV so prevalent in South Africa? Is it because of the way males are brought up to exert power and control over vulnerable women? South Africa is well known as a very patriarchal country and many cultural and traditional events and activities entrench this patriarchal behaviour reinforcing power over women. Media, in reporting incidences of GBV, focus on the victim by publishing headlines that read ‘a woman has been raped’ rather than stating ‘a man has raped a woman’. The issue of consent is blurred with Lobola where many wrongly assume that the woman now belongs to a man (a possession that he can use as he pleases). Consent is something that should be freely given, that can be recalled at any time and a person must be capable and understand the consequences of such consent. Young children, especially those below the age of 12 do not have the mental capacity for such consent. The South African Constitution clearly states that everyone is entitled to freedom and security of the person includes the right to be free from all forms of violence.^2,4,5^

Governments often lack the ability to address GBV, even where laws and codes of practice are in place mainly because it is tied up with gendered power relations that are deeply entrenched in some cultures. The lack of political and institutional will to deal with GBV is sustained by public attitudes. Gender-based violence is seen as part and parcel of life in many societies and there is little or no pressure on governments to address it.^2,4,5^

The justice and police systems shift blame to the victim rather than the perpetrator with questions posed such as ‘why were you alone’, ‘why were you dressed in that way’, ‘why were you out so late’. This automatically places vulnerable victims in a difficult situation when they actually need help and are survivors of horrific acts.

The World Bank states that GBV or violence against women and girls is a global pandemic that affects one in three women in their lifetime. The figures are staggering: 35% of women worldwide have experienced either physical and/or sexual intimate partner violence or non-partner sexual violence; 7% of women globally have been sexually assaulted by someone other than a partner and as many as 38% of murders of women are committed by an intimate partner, while 200 million women have been subjected to female genital mutilation. Violence and trauma are still some of the commonest reasons to seek healthcare in South Africa.^2,4,5^

Gender-based violence is not only devastating for survivors of violence and their families but also entails significant social and economic costs. The World Bank estimates that in some countries, violence against women costs countries up to 3.7% of their gross domestic product (GDP) – more than double what most governments spend on education.^2,4,5^

Gender-based violence, although very common in SA, is also present in other countries. There has been reports of violence against women at concerts in the United Kingdom, against women who defaecate in public in Bangladesh, against female university students in Norway and female genital mutilation in many countries. This emphasises the need to educate and involve men in all discussions and education to reduce this epidemic.^2,4,5^

Women’s rights campaigners have worked hard over many years to bring the issue of GBV to the attention of the world. This has led to a number of countries taking notable steps at national level to eradicate violence against women. The problem is that these steps have focussed primarily on improving the laws relating to violence against women but much less has been done to actually enforce these laws and to deal with the underlying cause of the problem which is the imbalance of power between women and men and the way gender roles are articulated at all levels of society.^2,4,5^

Although the president has spoken out against GBV and 16 days of activism has been set aside for GBV – this is insufficient. We as family physicians and the medical profession need to place more emphasis on teaching, training new doctors to be aware of this global epidemic and manage this scourge effectively. We also need to be more involved in community awareness programmes and empower communities to manage this epidemic which has been around long before the coronavirus disease 2019 (COVID-19) pandemic and will be around for more decades to come. We need to advocate at schools and universities to include managing, reporting and documenting GBV in our curriculum. The repercussions of GBV will be felt by our future generations as GBV has long-lasting mental and physical consequences and becomes a vicious cycle of abuse and gender-based violence.^2,4,5^
